# Extraosseous Plasmacytomas: A Radiologist’s Perspective—A Narrative Review of the Literature

**DOI:** 10.3390/diagnostics14161788

**Published:** 2024-08-16

**Authors:** Konstantinos Stefanidis, Gibran Yusuf, Francesk Mulita, Christos Tsalikidis, Athanasia Mitsala, Elissavet Konstantelou, Maria Kotsopoulou, Efstratios Koletsis, Michail Pitiakoudis, Platon Dimopoulos

**Affiliations:** 1 Radiology Department, Metaxa Cancer Hospital, 18537 Piraeus, Greece; kostef77@gmail.com; 2 Radiology Department, King’s College Hospital NHS Foundation Trust, London SE5 9RS, UK; gibran.yusuf@nhs.net; 3 Department of Surgery, General University Hospital of Patras, 75000 Patras, Greece; 4 Second Department of Surgery, University General Hospital of Alexandroupolis, Democritus University of Thrace Medical School, 68100 Alexandroupolis, Greece; ctsaliki@med.duth.gr (C.T.); nancymits20@gmail.com (A.M.); 5 Respiratory Department, Athens Naval Hospital, 11521 Athens, Greece; eliskonst@yahoo.gr; 6 Haematology Department, Metaxa Cancer Hospital, 18537 Piraeus, Greece; kotsopoulosmaria@yahoo.gr; 7 Department of Cardiothoracic Surgery, General University Hospital of Patras, 75000 Patras, Greece; ekoletsis@hotmail.com; 8 Department of Radiology, General University of Patras, 61000 Patras, Greece; dimopoylos.platonas@gmail.com

**Keywords:** extraosseous plasmacytomas, multiple myeloma, radiographic features

## Abstract

Extraosseous plasmacytomas (EPs) are rare neoplasms originating from plasma cells, often associated with multiple myeloma. EPs are classified into three subtypes: extramedullary myeloma, solitary extramedullary plasmacytoma (SEP), and multiple solitary plasmacytomas. They can manifest in various anatomical sites, including the lung, mediastinum, breast, liver, pancreas, stomach, mesentery, kidney, small and large bowel, testis, and soft tissue. Despite their rarity, EPs present a diagnostic challenge due to their non-specific imaging appearances, which can mimic other neoplastic and inflammatory conditions. This review aims to describe the radiographic features of EPs in the chest, abdomen, and pelvis based on a thorough analysis of the existing literature. While imaging plays a crucial role in the detection and characterization of EPs, histological confirmation is necessary to differentiate them from other neoplastic entities. The review underscores the importance of considering EPs in the differential diagnosis, particularly in patients with a history of multiple myeloma. Understanding the imaging characteristics of EPs is essential for accurate diagnosis and appropriate management. Early imaging is crucial in these patients to exclude the possibility of EP, as timely diagnosis can significantly impact patient outcomes.

## 1. Introduction

Plasmacytomas originate from the neoplastic proliferation of plasma cells, commonly occurring in patients with multiple myeloma. Plasmacytomas may either arise from the bone marrow, termed “Osseous Plasmacytomas”, or they may appear in anatomical sites unrelated to the bone marrow and non-contiguous with bone, termed “Extramedullary Myeloma” (EM) [1,2]. Extraosseous plasmacytoma (EP) is a localized plasma cell tumor outside the bone marrow, distinct from extramedullary myeloma (EM), which involves systemic disease [1,2,3]. EM can occur secondary to hematogenous spread [3].

Hematogenous spread to extramedullary plasmacytomas involves the migration of plasma cells from the bone marrow through the bloodstream to distant soft tissues, commonly seen in advanced multiple myeloma [2,3]. In contrast, primary extramedullary plasmacytomas arise de novo in soft tissues without prior bone marrow disease, driven by local factors rather than systemic dissemination [2,3].

However, plasmacytomas can also occur in the absence of systemic tumor involvement, defined as normal bone marrow aspirate, and in the absence of end-organ damage (hypercalcemia, renal insufficiency, and anemia), in which case they are termed “solitary plasmacytomas”. As previously described, solitary plasmacytomas may either be related to the bone [solitary osseous plasmacytoma] or may occur at extraosseous sites as “solitary extramedullary plasmacytoma” (SEP) [3]. A very rare subtype of plasma cell neoplasm called “multiple solitary plasmacytoma” (multiple-SEP) has been reported, which presents as multifocal sites of disease in the absence of any systemic involvement [3].

Solitary extraosseous plasmacytoma (SEP) is a special subtype of extramedullary plasmacytoma (EP), characterized by a single, localized tumor of plasma cells occurring in soft tissues outside the bone marrow without systemic involvement and tending to involve the liver, skin, kidneys, lymph nodes, and pancreas [3,4].

In contrast, EP is a broader term that encompasses any plasmacytoma occurring outside the bone marrow, including both isolated lesions and those associated with systemic plasma cell disorders such as multiple myeloma. Differentiating between SEP and the broader category of EP is crucial for accurate diagnosis, prognosis, and appropriate treatment planning [1,2,3,4].

To date, there has beena relative paucity of literature on the imaging appearances of extraosseous plasmacytomas. In this review, we aim to describe the radiographic features of extraosseous plasmacytomas in the chest, abdomen, and pelvis.

## 2. Lung

**Figure 1:**
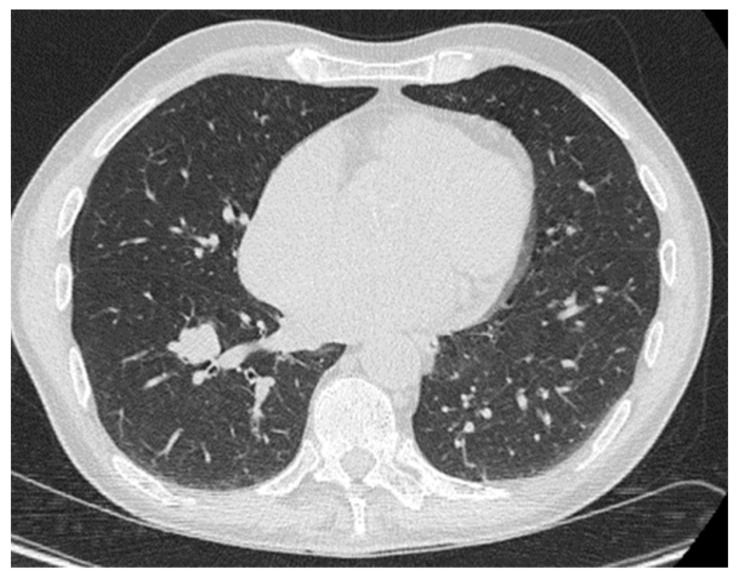
Biopsy-proven lung EP, case from our institution. Axial CT image demonstrates a lobulated lung nodule in the right lower lobe.

Extraosseous plasmacytomas rarely involve the lung, with limited cases in the literature. Most of the cases describe solitary extramedullary plasmacytomas as opposed to extramedullary myeloma [5,6,7,8,9,10,11,12,13]. Primary pulmonary plasmacytoma (PPP) is an uncommon form of extramedullary plasmacytoma, typically manifesting as a nodule or mass in the hilar regions. It is exceedingly rare for PPP to present with diffuse alveolar consolidation [14,15]. The radiographic imaging appearance of lung extraosseous plasmacytomas exhibits considerable variability, encompassing a spectrum of findings ranging from well-defined solitary masses to multifocal nodular lesions. Radiographic imaging appearances tend to be of a well-defined arterially enhancing hypodense soft tissue mass, without calcification or necrosis, devoiding a predominant pattern of specific localization (Figure 1) [5,9,12,16]. However, lung extraosseous plasmacytomas have a wide range of imaging appearances on CT and have even been described as presenting multiple lung nodules, making the diagnosis even more challenging [6,7,16]. Also, lung EP has been reported as an endobronchial mass, suggesting the consideration of lung EP in the differential diagnosis of endobronchial mass [16,17]. Both Tehari et al. and Lazerevic et al. have reported cases with diffuse infiltration of the lung, presenting as either consolidation or reticulonodular opacification [8,18].

Overall, imaging appearances of lung EP are non-specific with a wide differential, including primary lung neoplasm, metastasis, and infection. The comprehensive approach to diagnosis in cases of lung extraosseous plasmacytomas involves integrating various elements, including meticulous analysis of imaging modalities such as CT and PET/CT, thorough consideration of the patient’s clinical history, and the utilization of CT-guided biopsies for tissue sampling. However, perhaps the most crucial step in this diagnostic challenge is pathology confirmation, which provides definitive evidence and insight into the underlying histopathological features, guiding an accurate diagnosis and subsequent management decisions.

## 3. Mediastinum

Mediastinum extraosseous plasmacytomas represent a rare clinical entity that can sometimes be underdiagnosed. A mediastinal extraosseous plasmacytoma occurring in the posterior region is rare [19,20,21]. Mediastinum extraosseous plasmacytomas radiographic

**Figure 2:**
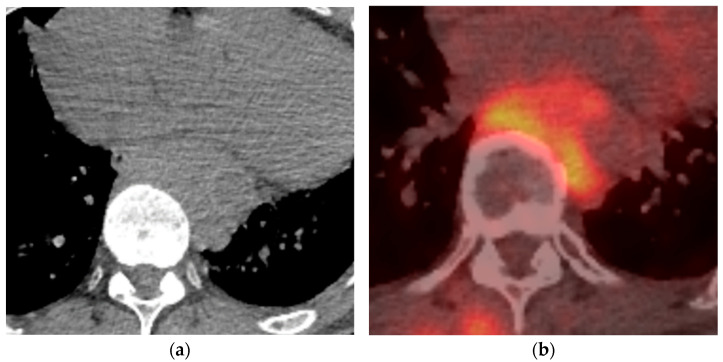
Biopsy-proven Mediastinal EP, case from our institution. (**a**) Unenhanced axial CT shows a posterior mediastinal soft tissue mass; (**b**) Axial PET-CT image shows heterogenous increased FDG-uptake in the posterior mediastinal mass.

**Figure 3:**
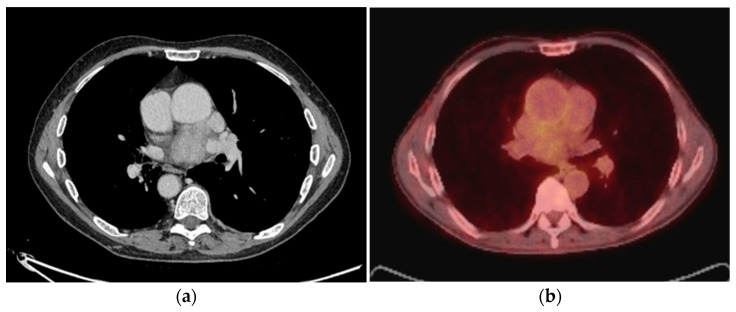
Biopsy-proven Mediastinal EP, case from our institution (**a**) Axial CT image shows an enhancing soft tissue lesion in the subcarinal space; (**b**) PET-CT image at the same level shows the mediastinal mass with mild heterogenous avidity.

Appearances most commonly present as a well-defined soft tissue mass with mild contrast enhancement and without vascular invasion (Figure 2 and Figure 3) [22,23,24,25,26]. Vascular invasion due to mediastinal extraosseous plasmacytomas has been infrequently documented in clinical literature, underscoring its significance in the comprehensive evaluation of these tumors. Despite its rarity, recognizing vascular invasion is paramount for precise staging, prognostic stratification, and informed treatment decision-making. Zhang et al. reported a remarkable case of mediastinal extraosseous plasmacytoma where the tumor not only occluded the right pulmonary artery but also invaded the superior vena cava. Despite the complexity of the condition, surgical resection was successfully performed. Subsequent follow-ups revealed no evidence of disease progression, and the patient remained alive [27]. Green et al. have also reported a heterogeneous mediastinal EP that was invading the right atrial and superior vena cava with extended multifocal extra-lung disease [28].

EPs originating intra-cardinally arean exceedingly rare occurrence. A case described by Vrettou et al. reported a PET avid and hypodense left ventricular mass that involved the intraventricular septum [29]. This had similar features to the case of a right atrium EP, described by Andrea et al. [30]. In both cases, the lesion was hypodense, well-defined, and hadno extra-cardiac extension.

The differential diagnosis for a well-defined soft tissue mass in the anterior mediastinum should include lymphoma, teratoma, and germ cell tumors. When it is located within the posterior mediastinum, the differential should include both lymphoma and neurogenic tumors.

From a radiological standpoint, diagnosing mediastinal extraosseous plasmacytomas presents a multifaceted challenge, demanding the amalgamation of various imaging modalities such as CT and PET/CT scans. This diagnostic journey, complemented by a meticulous review of clinical history and subsequent pathological validation, stands as a cornerstone for achieving precision in diagnosis. Given the propensity of mediastinal EP to involve vasculature, a heightened awareness of masses near blood vessels is warranted. Consequently, the judicious utilization of CT—angiography emerges as an indispensable tool for delineating vascular invasion, thereby offering crucial insights that not only confirm the diagnosis but also lay the groundwork for informed treatment strategies. This comprehensive radiological approach serves as a beacon, guiding clinicians toward tailored interventions and optimized patient outcomes.

## 4. Breast

Breast plasmacytoma (BP) is extremely rare. It can manifest either as a primary isolated tumor or as an extramedullary manifestation in multiple myeloma (MM) [31,32,33,34,35,36,37]. Despite the documentation of over 50 cases of EPs in the literature, there remains a scarcity of comprehensive descriptions regarding their imaging characteristics, leading to challenges in their accurate diagnosis. Most case reports depict sonographic features as heterogeneous, hypoechoic, hyper-vascular, and benign-appearing masses [38,39,40]. However, extraosseous plasmacytoma can also present as a hyperechoic, ill-defined lesion, albeit less commonly [41].

In the context of breast EP, mammographic appearances typically manifest as hyperdense oval or rounded masses with well-defined but irregular margins [27,28,29]. Notably, a rare case of extramedullary multiple myeloma has been documented, showcasing a hypoechoic well-defined mass in the breast and chest wall, further complicated by Sjogren’s syndrome [42]. In exceptional instances, diffuse infiltration of the breast may occur [39].

Additionally, breast EP has been reported as a palpable breast lump in patients with a history of multiple myeloma [43]. Given the broad spectrum of differential diagnoses for breast masses, encompassing fibroadenomas, lymphomas, and malignant neoplasms, among others, it is imperative to consider EP, particularly in cases with a history of multiple myeloma. A multidisciplinary approach involving radiologists, pathologists, and oncologists is essential for accurate diagnosis and appropriate management of breast EP, especially in the context of a complex clinical history.

From a radiological perspective, diagnosing breast extraosseous plasmacytomas presents significant challenges, as imaging findings on ultrasound and mammography are often nonspecific. Breast MRI has emerged as the preferred imaging modality due to its superior soft tissue contrast and sensitivity, particularly in detecting subtle abnormalities. With advancements in breast MRI technology, there is potential for identifying more characteristic radiographic features that could aid in the diagnosis of breast EP.

Moreover, performing biopsies of breast lesions under ultrasound guidance is a relatively straightforward procedure, facilitating the acquisition of tissue samples for pathological confirmation. This emphasizes the importance of integrating pathologic confirmation into the diagnostic process to establish standardized radiographic features of breast lesions based on pathology. Collaborative efforts between radiologists and pathologists are essential to correlate imaging findings with histopathological characteristics, ultimately enhancing diagnostic accuracy and informing appropriate management strategies for breast EP.

## 5. Liver

**Figure 4:**
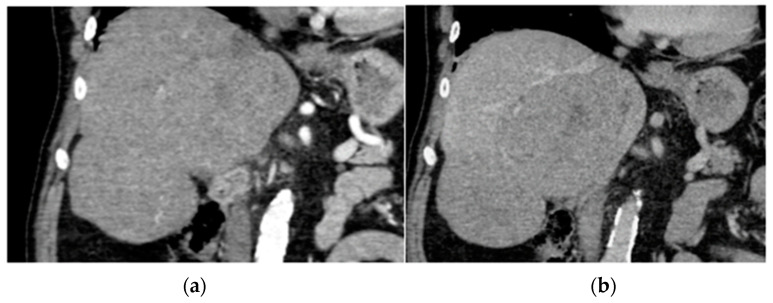
Biopsy-proven Liver EP, case from our institution (**a**) Arterial phase coronal CT shows two well-defined liver lesions, both of which show mild enhancement; (**b**) Portal venous phase coronal CT shows a very mild washout of contrast in both lesions.

Although EPs involving the liver are rare, their presence signifies a potentially more aggressive form of multiple myeloma, often necessitating chemotherapy or hematopoietic stem cell transplant [44]. The radiographic imaging appearances of hepatic MM manifesting in a focal or multifocal pattern are non-specific, with heterogeneous features described in a few published reports and case series to date [45,46,47,48,49,50,51,52,53]. Common ultrasound characteristics of EP in the liver typically manifest as a hypoechoic, well-defined mass; however, a target appearance with a hyperechoic center and hypoechoic rim has also been described [54,55]. The challenge in radiographic diagnosis lies in CT imaging appearances, where EP in the liver often presents as an arterially enhancing mass that can be either hypodense or isodense to the surrounding liver parenchyma on portal venous phase imaging (Figure 4) [54,55,56,57,58,59,60].

Given these imaging characteristics, the differential diagnosis for arterially enhancing liver lesions should encompass hepatocellular carcinoma, particularly in the context of cirrhosis, hypervascular metastasis, various subtypes of hemangioma, and mixed hepatocellular carcinoma with cholangiocarcinoma. Thorough evaluation combining imaging features, clinical context, and histopathological correlation is essential for accurate diagnosis and appropriate management of liver EP, especially considering its association with a more aggressive course of multiple myeloma. Collaboration between radiologists, oncologists, and pathologists is crucial to navigate the complexity of liver lesions and guide optimal treatment strategies.

In liver imaging, ultrasound serves as a valuable tool for detecting and characterizing lesions, providing initial insights into their morphology and composition. However, for a more comprehensive evaluation, CT and MRI scans play pivotal roles by offering detailed information about the vascularity of the tumor, particularly if it invades into vessels, and they can also detect smaller lesions that may not be adequately visualized on ultrasound alone. Additionally, PET/CT imaging can be particularly helpful in assessing tumor necrosis, providing valuable information about the metabolic activity of the lesions. By integrating these various imaging modalities, clinicians can obtain a comprehensive assessment of liver lesions, aiding in accurate diagnosis and guiding appropriate management decisions.

## 6. Pancreas

**Figure 5:**
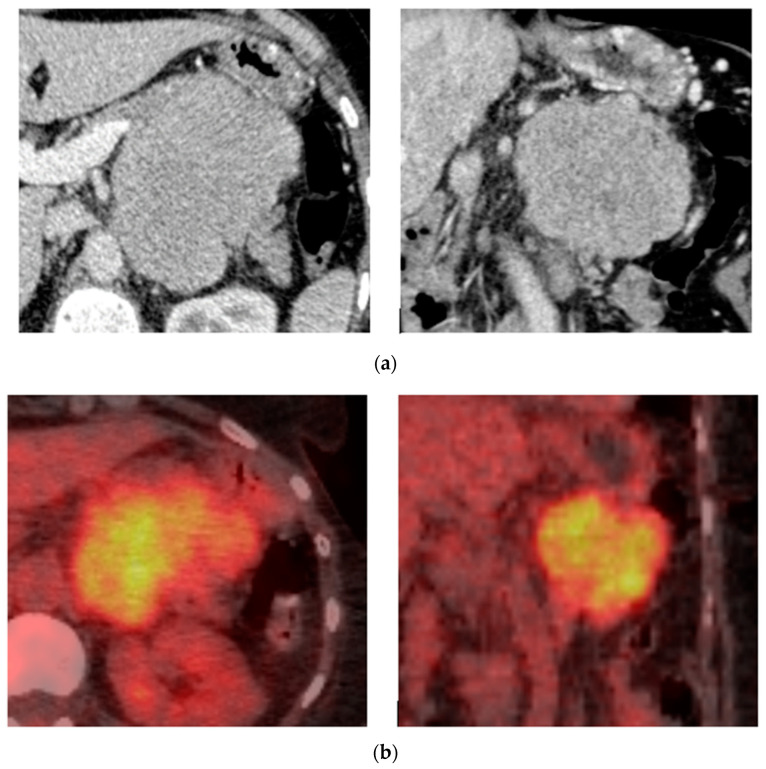
Biopsy-proven pancreas EP, case from our institution. (**a**) Post-contrast axial and coronal CT shows a well-defined homogenous lobular mass at the body of the pancreas; (**b**) Axial and coronal PET-CT shows increased FDG uptake in the pancreatic lesion.

**Figure 6:**
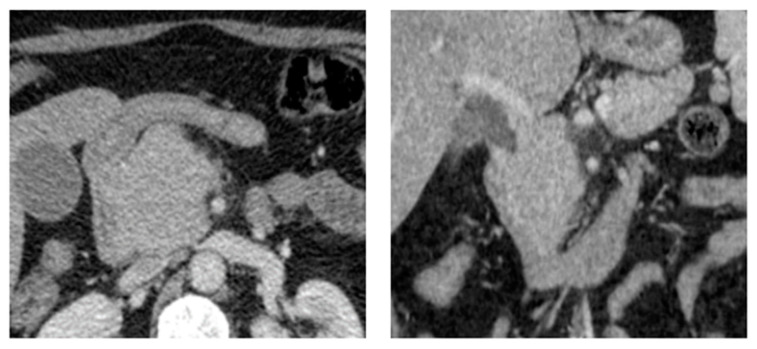
Biopsy-proven Pancreas EP, case from our institution. Axial and coronal post-contrast CT shows a well-defined homogenous mass at the pancreatic head. The coronal imaging demonstrates associated dilatation of the common bile duct.

The literature describing the radiographic imaging appearances of pancreatic EPs is indeed sparse, with reported findings spanning a wide spectrum. These encompass descriptions ranging from homogeneous or heterogeneous hypodense focal-enhancing lesions to diffuse infiltration of the pancreas [61,62,63,64]. In endoscopic ultrasound, pancreatic EP often presents as a hypoechoic heterogeneous mass, while PET-CT imaging typically reveals strong FDG uptake (Figure 5 and Figure 6) [62]. Additionally, associated features may include pancreatic duct dilatation, encasement of the celiac artery and portal vein, and infiltration of the superior mesenteric artery, with occlusion of the superior mesenteric vein, particularly in cases of large pancreatic head EP [65,66,67].

However, on imaging alone, differentiating EP from other enhancing pancreatic masses such as neuroendocrine tumors or hypervascular metastasis can be challenging. Nonetheless, a background of multiple myeloma can serve as a crucial clue, helping to refine the differential diagnosis and guide clinical decision-making. Collaboration between radiologists, oncologists, and pathologists is imperative to navigate the complexities of pancreatic lesions and optimize patient management strategies.

From the perspective of a radiologist, the pancreas poses challenges for visualization with ultrasound due to its retroperitoneal location. Therefore, the utilization of advanced imaging modalities such as CT and MRI scans becomes imperative to identify lesions with high diagnostic accuracy. Moreover, the administration of contrast during these scans helps us to characterize the vascularity of the tumor and assess for any vascular invasion, crucial factors in determining resectability and guiding treatment decisions.

It is essential to emphasize that while radiographic imaging plays a pivotal role in identifying and characterizing pancreatic lesions, the final diagnosis ultimately relies on pathological examination. Endoscopic ultrasound emerges as a valuable tool in this regard, as it not only offers radiological characterization but also provides the ability to obtain tissue biopsies, facilitating definitive diagnosis and informing subsequent management strategies.

In summary, the comprehensive evaluation of pancreatic lesions necessitates a multidisciplinary approach, wherein radiographic imaging serves as a vital component in conjunction with endoscopic ultrasound and pathological assessment. By leveraging the strengths of each modality, clinicians can achieve a more accurate diagnosis and effectively guide treatment interventions tailored to the individual patient’s needs.

## 7. Stomach

**Figure 7:**
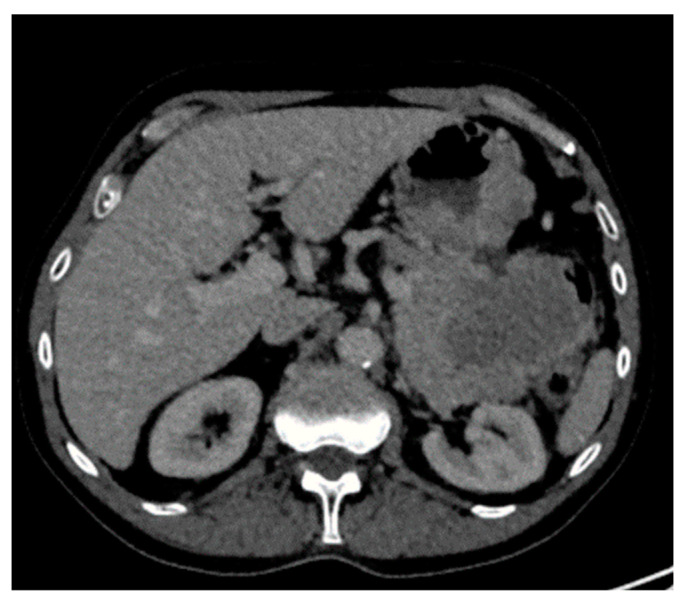
Biopsy-proven Gastric EP, case from our institution (Axial contrast-enhanced CT image shows a heterogeneous enhancing gastric mass with exophytic and intraluminal components.

EP involvement in the stomach is infrequent, and although over 100 cases have been described in the literature, there is very limited literature describing its imaging appearances [68]. The spectrum of presentation ranges from gastric wall thickening to an infiltrating vegetative mass with no specific pattern of enhancement (Figure 7) [69,70,71,72]. Radiographic imaging of the stomach EP can be challenging, especially when the stomach is not dilated. Diagnosis of gastric EP cannot be based solely on imaging findings, and the need for pathology and endoscopy is crucial.

Involvement of the stomach by EPs remains relatively infrequent, despite the sporadic documentation of over 100 cases in the medical literature [68]. However, the limited literature describing the imaging appearances of gastric EP underscores the intricacies involved in its diagnosis. The presentation spectrum of gastric EP is notably diverse, ranging from subtle gastric wall thickening to the manifestation of an infiltrating mass, often lacking a specific pattern of enhancement in imaging studies (Figure 7) [69,70,71,72]. Such variability in presentation poses challenges in the radiological evaluation of gastric EP, particularly when the stomach is not dilated or when lesions are subtle and challenging to discern.

In navigating the diagnostic landscape of gastric EP, it is paramount to recognize that diagnosis cannot be solely reliant on imaging findings alone. Rather, a comprehensive approach is imperative, necessitating collaboration between radiologists, gastroenterologists, and pathologists. Endoscopic evaluation holds pivotal significance, offering direct visualization of the lesion and facilitating targeted tissue biopsy for definitive diagnosis. Pathological examination of biopsy specimens serves as the cornerstone in confirming the diagnosis of gastric EP and distinguishing it from other gastric pathologies.

In conclusion, while radiographic imaging provides valuable insights into the presence and characteristics of gastric EP, its diagnosis requires a multidisciplinary effort. The integration of clinical, radiological, endoscopic, and pathological findings is indispensable for accurate diagnosis and optimal management of patients with gastric EP. This collaborative approach ensures comprehensive patient care and informed decision-making tailored to individual clinical scenarios.

## 8. Mesentery

The radiographic imaging appearances of mesenteric EP are reported either as thoseof a large, irregular enhancing mass with areas of central necrosis or as thoseof a well-defined, homogenous, mildly enhancing mass without central necrosis or calcification [73,74,75]. The reported size of mesenteric EP has been documented to exceed 10 cm in several case reports [74,76]. When these imaging appearances are encountered, the most likely diagnosis is a gastrointestinal stromal tumor orlymphoma. Nevertheless, EP should be considered in differential diagnosis when there is a history of multiple myeloma, histology is mandatory to determine the diagnosis.

The radiographic imaging appearances of mesenteric EPs present in two distinct patterns: one, a large, irregular enhancing mass with areas of central necrosis, and the other, a well-defined, homogeneous hypodense, mildly enhancing mass without central necrosis or calcification [73,74,75]. Notably, these mesenteric EP lesions can attain considerable size, documented to exceed 10 cm in several case reports, indicating their potential for significant enlargement [74,76].

While gastrointestinal stromal tumors and lymphoma are commonly considered in the differential diagnosis when encountering these imaging features, it is imperative to also consider EP, especially in patients with a history of multiple myeloma. Despite characteristic imaging findings, histological evaluation remains essential for definitive diagnosis. Collaboration between radiologists and pathologists is crucial in interpreting imaging findings and guiding appropriate diagnostic interventions for mesenteric EP.

Moreover, the role of PET/CT imaging cannot be overlooked, as it can aid in identifying these lesions even when they are small in size. PET/CT offers enhanced sensitivity in detecting metabolic activity, potentially allowing for early detection and intervention. Therefore, integrating PET/CT imaging into the diagnostic algorithm may further refine the evaluation of mesenteric EP, particularly in cases where conventional imaging modalities may be inconclusive [77]. This underscores the significance of a multidisciplinary approach and the evolving role of advanced imaging techniques in the comprehensive assessment of mesenteric EP.

## 9. Renal

The majority of the limited published articles have depicted renal EPs as non-enhancing or mildly enhancing, hyperdense renal masses without vascular invasion [78,79,80]. However, intriguingly, Todd et al. reported a case involving a mass originating from the renal pelvis that extended into the perirenal fat and appeared to involve the renal vein; although renal vein invasion was initially suspected, it was later excluded during surgery [60]. Furthermore, a case of primary renal EP was documented in a 14-year-old girl, wherein a CT scan revealed a homogeneous 3 cm mass with mild arterial enhancement. Following radical nephrectomy, the patient experienced no recurrence during 22 months of follow-up [78].

Despite these reported findings, the radiographic imaging appearances of renal EP remain nonspecific, often making it indistinguishable from renal cell or transitional cell carcinomas based on imaging alone. This highlights the challenge of establishing a definitive diagnosis solely through radiological means. Hence, a comprehensive diagnostic approach incorporating clinical history, imaging findings, and histopathological evaluation is crucial for accurate diagnosis and appropriate management. Collaboration between radiologists, urologists, and pathologists is essential in navigating the complexities of renal lesions and ensuring timely and tailored interventions for patients with suspected renal EP. Moreover, with advancements in imaging technology and the emergence of novel diagnostic modalities, such as molecular imaging techniques, there is hope for improved characterization and detection of renal EP with targeted molecular biomarkers, which may further enhance diagnostic accuracy.

## 10. Small and Large Bowel

EPs represent a rare subset of plasma cell neoplasms characterized by the aberrant proliferation of plasma cells outside the bone marrow. Their manifestation in the large bowel presents a distinct clinical entity, often marked by diffuse mural thickening secondary to a homogeneous hypodense soft tissue mass, frequently leading to luminal narrowing andobstructions [81,82]. Complications such as perforation and peritonitis can complicate the course of bowel EP, highlighting its clinical significance in early detection [83,84]. The evolution of advanced imaging modalities, notably CT enterography, holds promise foraugmenting diagnostic sensitivity for detecting these lesions.

**Figure 8:**
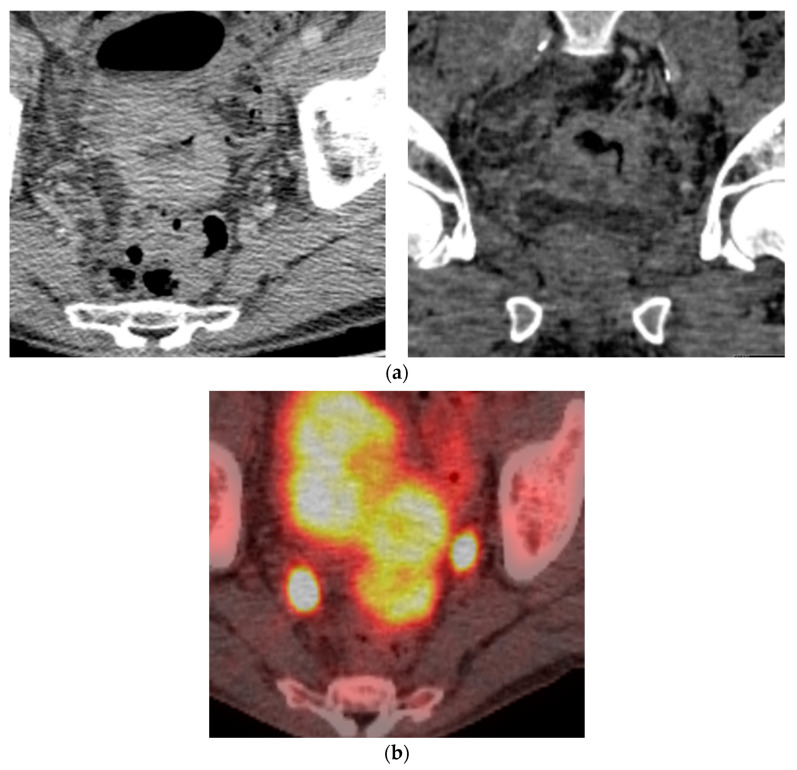
Biopsy-proven Colon EP, case from our institution. (**a**) Axial and coronal post-contrast CT demonstrates homogenous mural thickening of the sigmoid colon with resultant luminal narrowing; (**b**) Axial PET CT shows avid uptake in the sigmoid colon lesion.

A case encountered at our institution mirrors descriptions in the literature, illustrating a PET avid, circumferential, homogeneous soft tissue mass in the sigmoid colon with associated luminal narrowing (Figure 8). While colonic carcinoma typically dominates the differential diagnosis for such imaging presentations, consideration of colonic EP is imperative, particularly in patients with a history of multiple myeloma. The diagnosis of EP necessitates meticulous evaluation, often requiring a multidisciplinary approach involving radiologists, gastroenterologists, and pathologists.

Shifting the focus to the small bowel, EP manifests as a mural soft tissue mass with mild enhancement on CT imaging. Complications associated with small bowel EP encompass obstruction, perforation, and even intussusception secondary to small intraluminal nodules [85,86,87,88]. Despite not being the primary consideration in the differential diagnosis, which commonly encompasses adenocarcinoma and lymphoma, small bowel EP warrants consideration, especially in the appropriate clinical context. This underscores the importance of a comprehensive evaluation and a broad differential approach todiagnosing gastrointestinal EP lesions.

## 11. Testis

**Figure 9:**
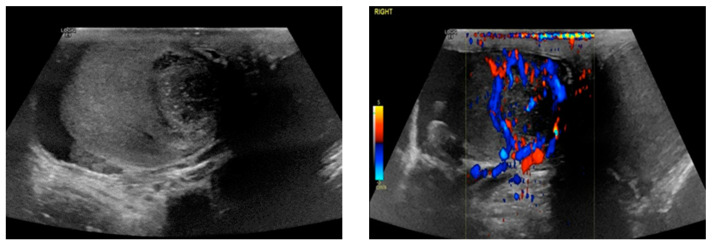
Biopsy-proven Testicular EP, case from our institution. Testicular ultrasound with B-mode and color Doppler shows a heterogeneous, hypoechoic lesion with punctuate calcifications and peripheral vascularity.

From a radiological perspective, the identification and characterization of testicular extraosseous plasmacytomaspose present unique challenges due to their rarity and variable presentations. Ultrasound imaging serves as the primary modality for initial evaluation, often revealing focal hypoechoic lesions with increased vascularity on duplex ultrasound, consistent with the most commonly reported appearance [89,90,91]. This is a very similar appearance to that of the case described at our institution, a heterogenous, hypo-reflective, hyperemic lesion (Figure 9). However, the heterogeneity of testicular EP is underscored by alternative presentations, such as grossly enlarged, heterogeneous, and hyperemic testicles, further complicating their radiological identification [92,93].

In clinical practice, differentiating testicular EP from other more prevalent testicular pathologies, including seminoma, germ cell tumor, or lymphoma, is paramount. This necessitates a nuanced approach that integrates imaging findings with clinical history and laboratory investigations. While testicular EP may reside at the lower end of the differential diagnosis list, its recognition remains crucial to avoid diagnostic oversight and ensure appropriate patient management.

Moreover, the inclusion of advanced imaging modalities, such as MRI and PET-CT, may offer additional insights into the extent of disease involvement and aid in treatment planning. Collaboration between radiologists, urologists, and oncologists is essential to navigate the complexities of testicular EP diagnosis and management effectively. By leveraging a multidisciplinary approach and remaining vigilant for atypical presentations, radiologists play a pivotal role in facilitating accurate diagnosis and optimal patient care in cases of testicular EP.

## 12. Soft Tissue

**Figure 10:**
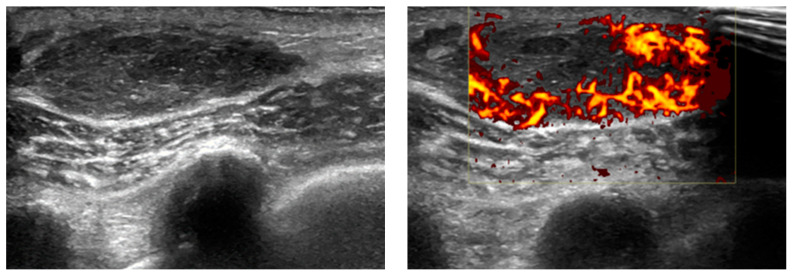
Biopsy-roven Soft tissue EP, case from our institution. Soft tissue ultrasound with B-mode and color Doppler shows a well-defined, heterogeneous, ovoid lesion with increased peripheral vascularity.

The described sonographic appearance of EPs in soft tissue is characterized by a well-defined, heterogeneous, hypoechoic reflective lesion with increased Doppler flow [94]. This observation closely aligns with a case documented at our institution (Figure 10). Despite their characteristic features, these radiographic appearances are nonspecific and may raise suspicion for alternative pathologies such as hematoma or primary neoplasm.

**Figure 11:**
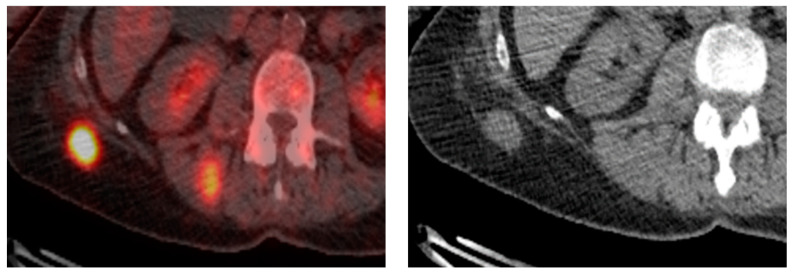
Biopsy-proven Soft tissue EP, case from our institution. Axial non-contrast CT and PET-CT show a well-defined, PET-avid soft tissue nodular lesion in the subcutaneous tissue.

In the context of cross-sectional imaging, EP typically manifests as a well-defined mass with increased tracer uptake on CT and PET-CT scans [95], akin to findings observed in a case encountered at our institution (Figure 11). While these imaging modalities provide valuable anatomical and functional information, they do not offer definitive diagnostic specificity for EP. Therefore, a comprehensive diagnostic approach is warranted, integrating clinical history, imaging findings, and histopathological evaluation to confirm the diagnosis.

The recognition of EP’s varied radiographic presentations underscores the importance of meticulous evaluation and a broad differential approach. Collaboration between radiologists, oncologists, and pathologists is indispensable in navigating the diagnostic challenges posed by soft tissue EP. By leveraging advanced imaging techniques and multidisciplinary expertise, clinicians can achieve accurate diagnoses and formulate tailored treatment strategies for patients with suspected EP.

Once a histological diagnosis of an EP has been established, it becomes imperative to assess for systemic involvement. This distinction is crucial for delineating between extramedullary plasmacytoma (EMP) and solitary plasmacytoma of Osseous (SPO), as it holds significant prognostic and therapeutic implications. EMP typically portends a poorer prognosis, characterized by an aggressive clinical course and an overall survival rate of 31%, contrasting starkly with the 59% survival rate observed in multiple myeloma localized solely to the marrow [96]. Given its classification as a high-risk multiple myeloma, EMP necessitates aggressive therapeutic interventions, including chemotherapy and potentially allogeneic transplantation [97]. In contrast, solitary plasmacytoma of Osseous (SP0) is associated with a more favorable prognosis, with localized radiotherapy yielding a remarkable 94% response rate [98].

Despite the clinical significance of distinguishing between EMP and SPO, both entities are exceedingly rare, collectively representing a small fraction of hematological malignancies. Multiple myeloma accounts for approximately 13% of all hematological malignancies, with approximately 4.8% of cases presenting with EMP at the time of diagnosis and an additional 3.4% developing EMP during the disease course [99]. The incidence of SEP is even lower, comprising only 1–3% of all plasma cell dyscrasias [100]. Consequently, the scarcity of literature describing their imaging appearances underscores the rarity of these conditions and emphasizes the need for early imaging evaluation to exclude the possibility of EP.

However, it is essential to acknowledge the limitations inherent in the existing body of literature. Much of the available evidence is derived from studies with low-level evidence, posing challenges in establishing robust conclusions. Additionally, the imaging characteristics of EP may overlap with those of various other radiographic conditions, further complicating the process of radiographic diagnosis. Despite these limitations, early recognition and accurate diagnosis of EP are pivotal for guiding appropriate therapeutic strategies and optimizing patient outcomes.

## 13. Conclusions

Our review not only highlights EPs’ wide array of presentations in different organs but also describes their typical imaging appearances. As the appearance of EP is non-specific, it is challenging to differentiate it from many other diagnoses, including primary neoplasms, metastases, or lymphoma. For this reason, it is important to consider EP as a diagnosis, especially in the context of multiple myeloma.
